# A *Trimethylguanosine Synthase1-like* (*TGS1*) homologue is implicated in vernalisation and flowering time control

**DOI:** 10.1007/s00122-021-03910-2

**Published:** 2021-07-13

**Authors:** Candy M. Taylor, Gagan Garg, Jens D. Berger, Federico M. Ribalta, Janine S. Croser, Karam B. Singh, Wallace A. Cowling, Lars G. Kamphuis, Matthew N. Nelson

**Affiliations:** 1 UWA School of Agriculture and Environment, The University of Western Australia, Perth, WA 6009 Australia; 2 The UWA Institute of Agriculture, The University of Western Australia, Perth, WA 6009 Australia; 3 Agriculture and Food, Commonwealth Scientific and Industrial Research Organisation, Floreat, WA 6014 Australia; 4 Centre for Crop and Disease Management, Curtin University, Bentley, WA 6102 Australia

## Abstract

**Key message:**

**A plant-specific** ***Trimethylguanosine Synthase1-like*** **homologue was identified as a candidate gene for the** ***efl*** **mutation in narrow-leafed lupin, which alters phenology by reducing vernalisation requirement.**

**Abstract:**

The vernalisation pathway is a key component of flowering time control in plants from temperate regions but is not well understood in the legume family. Here we examined vernalisation control in the temperate grain legume species, narrow-leafed lupin (*Lupinus angustifolius* L.), and discovered a candidate gene for an ethylene imine mutation (*efl*). The *efl* mutation changes phenology from late to mid-season flowering and additionally causes transformation from obligate to facultative vernalisation requirement. The *efl* locus was mapped to pseudochromosome NLL-10 in a recombinant inbred line (RIL) mapping population developed by accelerated single seed descent. Candidate genes were identified in the reference genome, and a diverse panel of narrow-leafed lupins was screened to validate mutations specific to accessions with *efl*. A non-synonymous SNP mutation within an S-adenosyl-L-methionine-dependent methyltransferase protein domain of a *Trimethylguanosine Synthase1-like* (*TGS1*) orthologue was identified as the candidate mutation giving rise to *efl*. This mutation caused substitution of an amino acid within an established motif at a position that is otherwise highly conserved in several plant families and was perfectly correlated with the *efl* phenotype in *F*_2_ and *F*_6_ genetic population and a panel of diverse accessions, including the original *efl* mutant. Expression of the *TGS1* homologue did not differ between wild-type and *efl* genotypes, supporting altered functional activity of the gene product. This is the first time a *TGS1* orthologue has been associated with vernalisation response and flowering time control in any plant species.

**Supplementary Information:**

The online version contains supplementary material available at 10.1007/s00122-021-03910-2.

## Introduction

Prolonged exposure to cold temperature represents an important stimulus for the promotion of flowering in many annual and biennial plants of temperate origins. This stimulus, known as vernalisation, is one of several environmental signals that are used by plants, together with endogenous cues, to align reproductive development with favourable biotic and abiotic conditions. The mechanisms through which plants perceive vernalisation are well-known to vary between families of plants (Bouché et al. 2017; Ream et al. 2012). Considerable progress has been made towards identifying the central integrator genes within the vernalisation pathway of cool-season legumes over the past decade. An *FT* orthologue (*LanFTc1*) was recently implicated as the main integrator gene for vernalisation response in narrow-leafed lupin (*Lupinus angustifolius* L.), and large deletions of up to 5162 bp within the promoter region of the gene were found to be responsible for the *Ku* and *Jul* phenotypes (Nelson et al. 2017; Taylor et al. 2019). Similarly, *FT* orthologues from the *FTa* clade have been identified as candidate/causal genes for major vernalisation-responsive QTLs in *Medicago truncatula* (Jaudal et al. 2013; Laurie et al. 2011), white lupin (*L. albus*) (Rychel et al. 2019), chickpea (*Cicer arietinum*) and its wild progenitor, *C. reticulatum* (Ortega et al. 2019).

In contrast, little is known about the identities and roles of upstream genes within the vernalisation pathway in legumes. Only one study to date has reported the discovery of an upstream gene within the vernalisation pathway in the Fabaceae. An orthologue of the *Vernalisation2* polycomb group protein gene (*MtVRN2*) which regulates *FT* in *Arabidopsis thaliana* (hereafter Arabidopsis) through chromatin modification (Gendall et al. 2001) was found to repress the expression of *MtFTa1* in *M. truncatula* (Jaudal et al. 2016). The molecular mechanisms through which this is achieved and whether this repression is direct or indirect remain unclear (Jaudal et al. 2016). The only other major finding regarding upstream genes has been the notable absence of well-known repressors of *FT* from the model Arabidopsis, such as *FLC*, in almost all legume species (Hecht et al. 2005, 2011), with the possible exception of soybean (*Glycine max*) (Jung et al. 2012).

Narrow-leafed lupin is an annual cool-season legume with a native distribution throughout the Mediterranean basin and Northern Africa (Gladstones 1970). It was domesticated as a pulse crop in the mid-twentieth century and has since become a valuable component of sustainable agricultural systems in Australia and northern European countries, such as Poland, Russia, and Germany (Hondelmann 1984; Lucas et al. 2015). This has largely been attributed to its capacity to improve soil fertility through symbiotic nitrogen fixation and the mobilisation of soil-bound phosphorus (Lambers et al. 2013), which, together with its traditional role as a break crop, enables the simultaneous benefits of reduced fertiliser input costs in addition to opportunities to reduce the severity of weeds, pests, and diseases that affect cereals (Harries et al. 2015).

Successful adaptation of annual plants to their environment depends on appropriate phenology (Nelson et al. 2010). The main strategy for adapting narrow-leafed lupins to short-season agricultural environments has been strong directional selection for early, vernalisation-insensitive phenology during breeding (Berger et al. 2012a, b). The dominant *Ku* early flowering time locus has been widely adopted throughout Australia to enable drought-escape in the warm Mediterranean climate experienced in the north of the Western Australia (WA) wheatbelt region, which to this day accounts for approximately 70% of Australian lupin production (ABARE 2018; Gladstones and Hill 1969). In parallel, the allelic *Julius* (*Jul*) locus was adopted in northern Europe for summer production and the timely maturation of crops before the onset of winter (Mikołajczyk 1966; Taylor et al. 2019).

The widespread use of *Ku* and *Jul* by breeders since their independent discoveries in the 1960s has drastically reduced phenological variation among elite varieties and limited the broader adaptive capacity of narrow-leafed lupin. Such varieties are not adapted to cool, longer season Mediterranean-type environments where partial vernalisation would be an advantage (Berger et al. 2012a, b). Modelling suggests that yield improvements of 13–16% (approximately 390–480 kg/ha) are possible in narrow-leafed lupin in long-season environments by delaying flowering two to three weeks relative to *Ku* varieties (Chen et al. 2017), reinforcing the need to increase the phenological diversity of the crop. Understanding the genetic background of flowering time is an important precursor for successfully incorporating novel phenological variation into elite domesticated varieties. In particular, knowledge of the vernalisation pathway is critical as it is the single most influential regulatory mechanism for flowering time control in narrow-leafed lupin crops (Gladstones and Hill 1969; Rahman and Gladstones 1972; Taylor et al. 2020a, b).

The *efl* locus represents a valuable yet under-utilised source of genetic variation for reduced vernalisation requirement in domesticated narrow-leafed lupin. It is a recessive mutation that was initially derived in the 1960s through mutagenesis of the Swedish variety, Borre, with 0.24% aqueous ethylene imine (Gladstones 1977). The mutation causes a shift from an obligate to facultative vernalisation requirement, where vernalisation accelerates the transition to reproductive growth but is no longer essential (Gladstones 1982; Landers 1995). The resulting flowering time phenotype is roughly 10–14 days earlier than *ku* wild types and two to three weeks later than *Ku* vernalisation-insensitive types under long-season environmental conditions (Gladstones 1982). Although the first *efl* variety, named Chittick, was believed to have a phenology close to the optimal for many mid-high rainfall environments at the time of its release in the 1980s (Gladstones 1982), it was outcompeted by earlier flowering *Ku* varieties with important agronomic advances, including resistance to phomopsis stem and pod blight (*Diaporthe toxica*) (Cowling et al. 1988, 1987; Cowling and Wood 1989). Since then, *efl* has only been bred into one other Australian variety, Wandoo (Cowling 1999; Cowling 2020), and its integration into modern breeding lines alongside other key traits would be aided by molecular markers to improve the efficiency of selection.

Here we report the discovery of a plant-specific *Trimethylguanosine Synthase1-like* (*TGS1*) orthologue as a strong candidate gene for *efl* and propose that a G/A SNP transition within its coding sequence (CDS) produces an amino acid substitution that interrupts a conserved motif critical for substrate binding. *TGS1* has significant roles in post-transcriptional regulation and processing of a wide variety of RNA (e.g. small nuclear (sn) RNA, small nucleolar (sno) RNA, telomerase RNA and selenoprotein mRNAs) across all kingdoms (Chen et al. 2020; Girard et al. 2008; Mouaikel et al. 2003, 2002; Wurth et al. 2014). This is the first time a *TGS1* homologue has been linked to vernalisation response within the plant kingdom.

## Methods

### Genetic population development

*F*_2_ and *F*_6_ bi-parental mapping populations were developed from a cross between narrow-leafed lupin varieties, Chittick (maternal parent) and Geebung (paternal parent). Chittick is homozygous for the *efl* mutation (Gladstones 1982) and has a facultative vernalisation requirement, while Geebung has an obligate vernalisation requirement (Berger et al. 2012a). Geebung has a putative *Efl* genotype and was chosen as the late-flowering parent in this RIL population because it has a lower coefficient of co-ancestry with Chittick than other late-flowering varieties (e.g. Marri, Uniharvest, and Uniwhite) on the basis of pedigree information (Cowling 2020) and Euclidean distances based on > 10,000 DArTseq SNP markers (Mousavi-Derazmahalleh et al. 2018).

Seeds of Chittick and Geebung were germinated on Petri dishes lined with moistened filter paper and vernalised in the dark at 4 °C for 21 days. Seedlings were then planted in steam-pasteurised soil mix (UWA Plant Bio Mix, Richgro Garden Products Australia Pty Ltd) in 7 × 7 cm (750 mL) pots and grown at 20 °C with natural photoperiod (ranging 10–12 h) in a phytotron at The University of Western Australia (UWA), Perth, Australia (31°59′04.8" S, 115°49′09.5" E), in 2016. Plants were watered daily and fertilised fortnightly with water-soluble NPK fertiliser with micronutrients (Peters Excel, Everris NA Inc.) at a rate of 250 mg/pot. After crossing and maturation of hybrid seed, one *F*_1_ seed was germinated, vernalised, and grown in a pot in the phytotron as described above. PCR-based assays of the LaIND_026 molecular marker (5’ GGACCATTGTGCCTTGGG 3’; 5’ GAAGTGGCAGAACTGGTTGG 3’) (Kamphuis et al. 2015), for which Chittick and Geebung have contrasting alleles (~ 200 and ~ 180 bp amplicons, respectively), were used to confirm this plant was a true *F*_1_ hybrid (data not shown). More than 550 *F*_2_ seed were harvested from this *F*_1_ individual at maturity.

An accelerated single seed descent (aSSD) approach utilising an optimised ratio of red to far-red light for legumes (Croser et al. 2016) was used to rapidly progress 330 *F*_2_ individuals to the *F*_6_ generation. In each selfing generation, seeds were firstly germinated in Jiffy-7® pellets and vernalised at 4 °C for 21 days to satisfy the requirements of obligate vernalisation genotypes, thereby ensuring rapid and synchronised flowering and seed production in all RILs. Seedlings were then transplanted to 750-mL pots and cared for as described above. Plants were grown at 24 °C (day)/20 °C (night) under natural light supplemented with light-emitting diode arrays (AP67, L-series, Valoya® lights, Helsinki, Finland) to create a 20-h photoperiod (Croser et al. 2016). Four generations of aSSD occurred over a period of 13 months (average generation time of 13.5 weeks, inclusive of vernalisation treatment), and a total of 185 *F*_5_-derived *F*_6_ RILs were harvested.

### Phenotyping F_2_ and F_6_ mapping populations

Segregation of flowering time was assessed in 200 *F*_2_ individuals, 10 *F*_1_ hybrids, and 20 biological replicates of each parent (Chittick and Geebung) from mid-May to late-August 2017 in a phytotron located at UWA. This trial occurred in parallel to the *F*_6_ recombinant inbred line (RIL) population development using separate *F*_2_ individuals. Seeds were germinated in Jiffy-7® pellets and vernalised for 40 days in the phytotron at 15 °C. This vernalisation treatment was designed to enhance the difference in flowering time between Chittick and Geebung under mild vernalisation conditions (JD Berger, unpublished data). Seedlings were transplanted into pots following vernalisation treatment (as described above) and placed within a randomised block design. The temperature of the phytotron was subsequently increased to 21 °C to prevent further vernalisation. Flowering was scored when the first flower was fully opened (i.e. displayed an erect standard petal) or displayed a deepening pink pigmentation on the petals, both of which are indicative of anthesis (Dracup and Kirby 1996). A Chi-square goodness-of-fit test was used to evaluate whether segregation of *efl*:*Efl* phenotypes conformed to the expected 1:3 ratio for a Mendelian gene in an *F*_2_ population.

The *F*_6_ RIL population was similarly phenotyped in a phytotron at UWA under natural daylight (ranging from 10 to 11.5 h) in 2018. Three *F*_6_ seeds per RIL (i.e. biological replicates) were sown if sufficient seeds were available; however, 17 RILs had sufficient seed for only one or two biological replicates (Online Resource 1). Biological replicates of Chittick (*n* = 3) and Geebung (*n* = 12) were also phenotyped. Germination, vernalisation, transplanting, and phenotyping within an incomplete block design were as described for the *F*_2_ population. Each RIL was assigned to a phenotypic category based on the average flowering time of biological replicates (the ‘*efl*’ group with similar flowering to Chittick and the ‘*Efl*’ phenotype group with similar flowering time to Geebung), and a Chi-square goodness-of-fit test was used to evaluate the expected 1:1 segregation of *efl*:*Efl* phenotypes in this RIL population.

### Genotyping the ***F***_6_ RILs population and its parents

#### DArT-seq genotyping of the ***F***_6_ population and its parents

Freeze-dried leaf material from the 185 *F*_6_ RILs and population parents was provided to Diversity Arrays Technology Pty Ltd (Canberra, Australia) for gDNA extraction and subsequent DArTseq™ genotyping-by-sequencing (hereafter DArTseq) (Sansaloni et al. 2011), as described by Mousavi-Derazmahalleh et al. (2018), to produce SNP and presence/absence variation (PAV) markers. The physical coordinates of all SNP and PAV markers were determined by BLAST of the DArTseq marker sequence to the Tanjil reference genome assembly (Hane et al. 2017) using a minimum sequence identity threshold of 80% and an e-value of 5e − 07.

#### Whole-genome re-sequencing of Chittick and Geebung

DNA of Chittick and Geebung was extracted from young leaves using a CTAB method (Doyle and Doyle 1990). DNA samples were then submitted to Novogene (Hong Kong) for 2 × 150 bp Paired-End library preparation using the default recommendations in the NEBNext® DNA Library Prep Master Mix Set for Illumina® kit and whole-genome re-sequencing at approximately 10 × coverage using Illumina® sequencing by synthesis technology. Raw reads were checked for quality and adapter content using FastQC (Andrews 2010). Adapter trimming was carried out using cutadapt (parameters: -format fastq -overlap 10 -times 3 -minimum-length 25) (Martin 2011). The raw sequencing data generated have been deposited in the Sequencing Read Archive (SRA; BioProject ID PRJNA674415).

#### Genotyping the *LanFTc1* promoter region in Chittick and Geebung

Whole-genome re-sequencing reads for Chittick and Geebung were aligned to the wild-type (*ku*) *LanFTc1* promoter sequence from accession P27255 (GenBank ID KT862491) (Nelson et al. 2017). This alignment was used to establish the *LanFTc1* promoter genotype of Chittick and Geebung and confirm whether the *efl* locus was distinct from a series of large deletions within this region that underlies the *Ku* and *Jul* alleles for early flowering time in domesticated narrow-leafed lupin.

### Linkage and QTL mapping

SNP and PAV DArTseq markers were filtered for quality control purposes. Markers with > 50% call rate, a homozygous allele frequency between 0.2 and 0.8 (SNPs only), and a Chi-square *P* value > 1e − 05 were retained. SNPs with > 15% heterozygosity were additionally removed. All markers were then phased according to the parental genotypes, and any markers with an ambiguous phase were represented in both phases. A qualitative marker representing the *efl* phenotype was created for the purposes of linkage map construction and QTL mapping. Homozygous *efl* genotypes were assigned to *F*_6_ RILs with an early average flowering time of 71–78 days, while homozygous *Efl* genotypes were assigned to late-flowering time averages of 84–95 days. Missing values were allocated to RILs with intermediate phenotypes (79–83 days to flowering) (Online Resource 1). The qualitative *efl* phenotype-derived marker and SNP/PAV markers were encoded in the same way with ‘*A*’ representing the maternal *efl* genotype, ‘*B*’ representing the paternal *Efl* genotype, and ‘-’ representing missing or heterozygous values.

A linkage map for the *F*_6_ RIL population was created using the ASMap (Taylor and Butler 2017) and Rqtl (Broman et al. 2003) packages for *R* computing software. The following settings were implemented: the Kosambi method for genetic distance calculation; a count function to minimise the sum of recombination events; a *P* value threshold of 1e − 18 for clustering markers; and a maximum of 15 cM between linked markers. Marker genotyping for three *F*_6_ RILs (CxG_F6_100, CxG_F6_101, and CxG_F6_155) was identified as problematic based on the high frequency of missing values (*n* > 650) and double crossovers they introduced (*n* = 16 to 28), and was therefore removed from further analysis. Two *F*_6_ RILs (CxG_F6_215 and CxG_F6_218) had 98% similarity in genotype; these two RILs were recorded as a single consensus genotype. Clusters with ≤ 5 markers were discarded, as were linkage groups comprising only ambiguously phased markers whose respective mirrored partners mapped appropriately within larger linkage groups.

QTL mapping was conducted within the ASMap and Rqtl packages (Broman et al. 2003; Taylor and Butler 2017). An interval mapping approach implementing the expectation–maximisation (EM) algorithm was used to detect QTLs for mean days to flowering in the *F*_6_ RILs. A genome-wide logarithm of the odds (LOD) threshold of 2.95 was used to declare QTL at a 95% confidence interval. This threshold was established via permutation testing with 1000 permutations.

### Defining the *efl* locus and its candidate genes

FGenesh (v2.6) (Solovyev et al. 2006) was used to identify predicted open reading frames (ORF) within a mapped region of interest sourced from an improved PacBio assembly for Tanjil (G Garg, LG Kamphuis, R Foley & K Singh, unpublished data). Organism-specific gene-finding parameters were implemented for *Glycine max*, which was selected as the closest legume relative of narrow-leafed lupin. Sequencing reads from Chittick, Geebung, and additional 43 accessions (G Garg, LG Kamphuis, R Foley & K Singh, unpublished data) were aligned to the region of interest using Bowtie (v2.3.4) (Langmead and Salzberg 2012), and variants were called using SAMtools (Li et al. 2009). The predicted CDS of a candidate gene was used as a query sequence for BLASTn against the NCBI non-redundant (nr) nucleotide sequence database to determine a putative functional annotation. Phylogenetic analysis of the candidate gene family was conducted via CLUSTAL Omega (v1.2.4; https://www.ebi.ac.uk/Tools/msa/clustalo/) alignment of protein sequences from narrow-leafed lupin, 16 other papilionoid legumes, 12 diploid species from five other angiosperm families, and yeast (*Saccharomyces cerevisiae*) (Online Resource 2). Protein sequences for the phylogenetic analysis were identified through BLASTp against the NCBI and Unité de Recherche Génomique Info (URGI) non-redundant protein sequence databases. Based on this alignment, a neighbour-joining phylogenetic tree without distance corrections was constructed using CLUSTAL Omega and visualised using FigTree (v1.4.3).

### Validating an *efl* candidate gene and putative causal SNP

#### Co-segregation of the *efl* phenotype and SNP mutation

PCR primers (5’ CATCTGTAGTGGCAATGCGC 3’ and 5’ AAGGCCTTTGCTCACTCCAA 3’) were designed to amplify a 567-bp gDNA fragment within the *efl* candidate gene in which a unique SNP of interest with a G/A transition was approximately central. PCR amplification was conducted according to reaction and cycling programme details provided in Online Resource 3. PCR amplicons were submitted to Macrogen (Seoul, South Korea) for bi-directional Sanger sequencing. SNP genotypes were tested for co-segregation with *efl* phenotype in the *F*_2_ and *F*_6_ bi-parental populations and a diverse set of 40 wild and 49 domesticated accessions (Online Resources 4 and 5).

#### Predicted protein composition, domains, and structure

Haplotypes for the candidate gene CDS were established by comparison of SNP genotypes observed within Chittick, Geebung, Tanjil, 43 accessions with re-sequencing data, and three other key narrow-leafed lupins, including: N4229 (the original *efl* mutant), Borre (the variety used in mutagenesis to derive *efl*), and Wandoo (the only other commercial variety with *efl*). SNPs within the CDS of these latter three accessions were genotyped via bi-directional Sanger sequencing (Macrogen: Seoul, South Korea) of five PCR amplicons targeting polymorphic exons within the candidate gene (Online Resources 3 and 6). Translated amino acid sequences for each unique haplotype were aligned using CLUSTAL Omega (v1.2.4; https://www.ebi.ac.uk/Tools/msa/clustalo/) and were examined in InterPro Scan (https://www.ebi.ac.uk/interpro/result/InterProScan/) to determine whether any substitutions interrupted functional protein domains. Finally, 3D protein models were constructed based on the 3gdh.1.A template using the SWISS-MODEL automated protein structure homology-modelling server (https://swissmodel.expasy.org/).

#### Gene expression analyses

Seeds of Chittick and Geebung were imbibed in Milli-Q water for 6 h and were either immediately sown (non-vernalised treatment) or incubated in Petri dishes at 4 °C for 21 days (vernalisation treatment) before sowing. Imbibition was staggered so that the vernalised treatment had accumulated approximately the same thermal time (expressed as the number of degree-days with a 0 °C baseline temperature) as the non-vernalised treatment at the time of sowing. All plants were grown in a phytotron at UWA under natural daylight (11.5–13.75 h). An average diurnal ambient temperature regime of 21 °C (day) and 18 °C (night) was used to prevent further vernalisation. The four uppermost fully emerged leaves were snap-frozen in liquid nitrogen when plants had reached the 4-, 8-, 12-, and 16-leaf stages and at flowering (in the case of vernalised plants) or when the primary inflorescence began to demonstrate signs of floral meristem reversion (in the case of non-vernalised plants).

RNA extraction, cDNA synthesis, and qPCR analyses were performed on three biological replicates according to the methods described by Taylor et al. (2016). Relative transcript abundance of the candidate gene was measured as the average threshold cycle (*C*_*T*_) based on two primer pairs specific to different exons within each gene (Online Resource 7) and was subsequently normalised against the previously validated reference gene, *Ubiquitin C* (*UBC*) (Taylor et al. 2016). The final relative expression of the candidate gene was expressed as 40-Δ*C*_*T*_. A value of 40 on this log_2_ scale is equivalent to the expression of the reference gene, while the raw *C*_*T*_ value of the reference gene reflects the minimum value on the scale. ANOVA and Tukey HSD post hoc statistical tests were conducted on the 40-Δ*C*_*T*_ values within *R* software.

## Results

### Flowering time distribution in bi-parental mapping populations

**Fig. 1:**
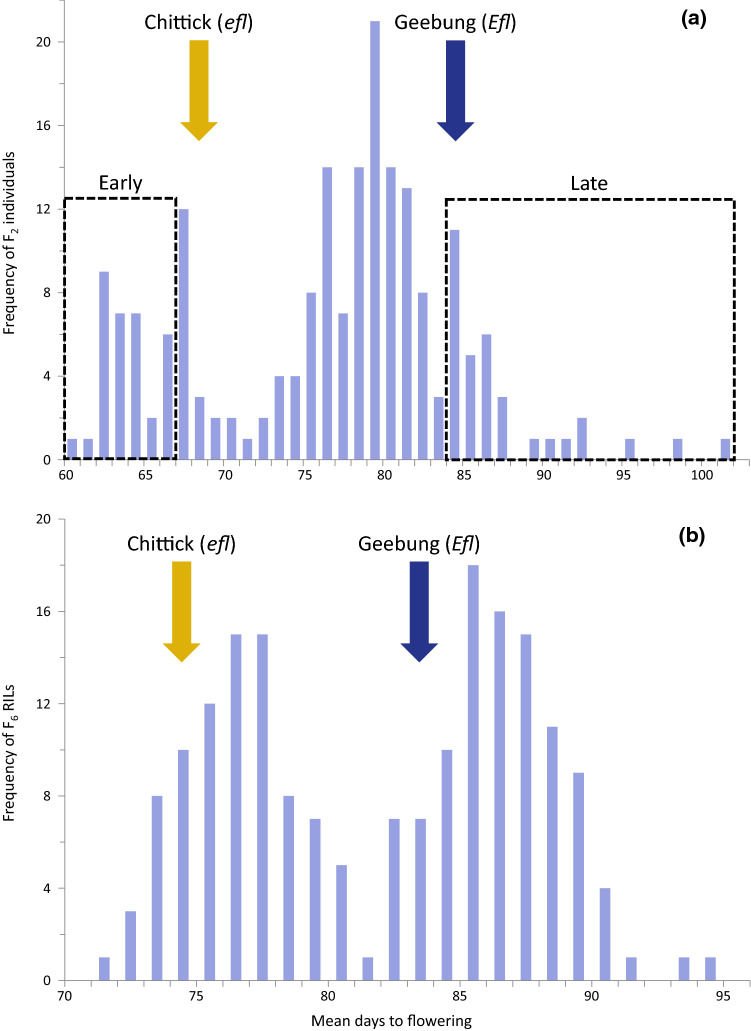
Frequency of mean days to flowering for **a** 200 *F*_2_ individuals and **b** 185 *F*_6_ recombinant inbred lines of narrow-leafed lupin (*Lupinus angustifolius* L.) segregating at the *efl* flowering time locus. The populations were derived from a single cross between varieties, Chittick (*efl*) and Geebung (*Efl*). The mean flowering times for the parental varieties are indicated by yellow and dark blue arrows, respectively. Dashed boxes in (a) highlight the 33 earliest and 32 latest flowering *F*_2_ individuals whose DNA were combined into early and late bulk samples for genotyping

Bi-parental mapping populations designed to study *efl* were developed from Australian narrow-leafed lupin varieties, Chittick (maternal parent; *efl* genotype) and Geebung (paternal parent; *Efl* genotype). Chittick flowered on average 15.6 days earlier than Geebung during the first phenotyping trial evaluating *F*_1_ and *F*_2_ progeny (Fig. 1a). Consistent with expectations for a recessive gene with Mendelian inheritance, the *F*_1_ hybrids had an average flowering time equal to Geebung at 84.5 days and early (*efl*-like) to late (*Efl*-like) flowering times segregated in an approximate 1:3 ratio among *F*_2_ individuals (*χ*^2^[DF = 1, *n* = 198] = 0.17, *P* > 0.05).

A difference of 9.1 days to flowering separated Chittick and Geebung in the second phenotyping trial evaluating the *F*_6_ RIL population. Average flowering times in the 185 *F*_6_ RILs followed a bimodal distribution with the two modes centred on the two parents (Fig. 1b). For the purposes of statistical testing, RILs with 71–81 mean days to flowering were assigned to the *efl*-like phenotypic category (*n* = 87), and RILs with 82–93 mean days to flowering were assigned to the *Efl*-like phenotypic category (*n* = 98). The number of progeny in each category conformed to the expected 1:1 ratio of *efl*:*Efl* (*χ*^2^[DF = 1] = 0.538, *P* = 0.463).

### Linkage and QTL mapping localises *efl* to a 267-Kb region on NLL-10

DArTseq genotyping of the *F*_6_ RIL population and its parental varieties produced 2503 SNP and 17,710 PAV molecular markers. Following stringent quality filtering and the mirror-phasing of 355 ambiguous markers, a total of 1248 SNP and 1473 PAV markers, plus one qualitative marker representing the *efl* phenotype, were retained for linkage mapping. This filtered set of markers adequately represented all pseudochromosomes except for NLL-11, NLL-15, and NLL-19, which each had five or less markers (Online Resource 8).

The preliminary linkage map based on the *F*_6_ RIL population comprised 76 linkage groups, which far exceeded the number of haploid chromosomes (*n* = 20) for narrow-leafed lupin. Twenty-seven of these linkage groups were made up almost exclusively of ambiguously phased markers whose mirror partners mapped well in larger linkage groups. As a result, these artefactual linkage groups were discarded along with 10 small clusters with fewer than five loci.

The final linkage map incorporated 1035 SNP, 1279 PAV markers, and the *efl* phenotypic marker among the 181 *F*_6_ RILs (Online Resource 9). A total of 23 linkage groups were constructed, which largely represented 17 of the 20 haploid chromosomes for narrow-leafed lupin (Table 1). The size of individual linkage groups ranged from 0.6 to 131.8 cM, producing an overall map size of 781.2 cM.

**Table 1:** Summary of the 23 linkage groups constructed in a genetic map of an *F*_6_ RIL population (*n* = 181) derived between Australian narrow-leafed lupin (*Lupinus angustifolius* L.) varieties, Chittick (*efl*, *ku*), and Geebung (*Efl*, *ku*)

Pseudochromosome represented by linkage group	Linkage group ID	Size of linkage group (cM)	Number of markers in linkage group
NLL-01 (top)	CxG_LG01	27.7	43
NLL-01 (bottom)	CxG_LG02	0.6	10
NLL-02	CxG_LG03	80.7	227
NLL-03 (top)	CxG_LG04	1.7	12
NLL-03 (bottom)	CxG_LG05	86.3	188
NLL-04 (top)	CxG_LG06	41.5	80
NLL-04 (bottom)	CxG_LG07	23.3	43
NLL-05	CxG_LG08	26.4	86
NLL-06 (top)	CxG_LG09	6.4	21
NLL-06 (bottom)	CxG_LG10	44.3	342
NLL-07	CxG_LG11	17.5	84
NLL-08	CxG_LG12	8.0	24
NLL-09	CxG_LG13	77.2	92
NLL-10	CxG_LG14	131.8	403
NLL-11	*		
NLL-12 (top)	CxG_LG15	4.4	12
NLL-12 (bottom)	CxG_LG16	12.5	55
NLL-13	CxG_LG17	7.1	27
NLL-14	CxG_LG18	75.0	198
NLL-15	*		
NLL-16	CxG_LG19	26.4	72
NLL-17 (top)	CxG_LG20	5.5	36
NLL-17 (bottom)	CxG_LG21	0.7	8
NLL-18	CxG_LG22	67.1	222
NLL-19	*		
NLL-20	CxG_LG23	9.1	30
Total		781.2	2315

The genetic map incorporated 2314 DArTseq molecular markers and one artificial marker representing *efl* phenotype within the RIL population developed in this study

Asterisks (*) denote incidences where a linkage group was not found to largely represent one of the 20 narrow-leafed lupin pseudochromosomes (Hane et al. 2017)

Interval mapping resolved a major QTL for flowering time on the CxG_LG14 linkage group corresponding to pseudochromosome NLL-10. The QTL explained 81.95% of phenotypic variation for flowering time within the *F*_6_ RIL population and achieved a maximum LOD score of 67.25, which was centred on the qualitative *efl* phenotypic marker (Online Resource 9). The two DArTseq markers flanking the *efl* phenotypic marker (LangCGRIL_PAV04800, LOD 63.91; LangCGRIL_SNP1280, LOD 58.63) were separated by a 1.26 cM genetic interval. However, a physical interval for the *efl* locus could not be initially established as LangCGRIL_PAV04800 was placed on NLL-06 (Online Resource 9), which suggested an assembly error within the region of interest in the published Tanjil reference genome (Hane et al. 2017). To overcome this apparent assembly error issue, sequences from the LangCGRIL_PAV04800 and LangCGRIL_SNP1280 DArTseq markers were physically located within an improved PacBio assembly for Tanjil (G Garg, LG Kamphuis, R Foley, and K Singh, unpublished data). Both markers were located within a single contig from this assembly and consequently enabled a 267,160-bp physical region of interest to be defined (GenBank ID MW218472).

### *efl* is not conditioned by a deletion in the *LanFTc1* promoter

*LanFTc1* is a highly important central regulator of the vernalisation pathway in narrow-leafed lupin. To eliminate the possible involvement of *LanFTc1* (NLL-10: 8,016,834–8,033,155), which is located approximately 2.5-Mb downstream of the *efl* locus NLL-10, we compared the 5’ regulatory region of *LanFTc1* in Chittick and Geebung. Specifically, we sought determine whether both varieties share the wild-type *LanFTc1* promoter (*ku*) or whether Chittick possesses one of three large deletions (1208 bp; 1423 bp, *Ku*; and 5162 bp, *Jul*), which are the only known polymorphisms associated with de-repressed *LanFTc1* expression, reduced vernalisation response, and early flowering time (Nelson et al. 2017; Taylor et al. 2019). High-throughput Illumina re-sequencing produced 42,338,865 (~ 13 × depth coverage) and 34,313,565 (~ 11 × depth coverage) short reads for Chittick and Geebung, respectively, which were mapped to the wild-type (*ku*) *LanFTc1* promoter sequence (GenBank ID KT862491). Both parents shared the *ku* wild-type allele, confirming that the *efl* phenotype is derived from an independent gene located upstream of *LanFTc1* on NLL-10.

### *Trimethylguanosine Synthase1-like* (*LanTGS1*) as a candidate gene for *efl*

#### Discovery of a strong positional candidate gene and underlying mutation

The 267,160-bp region of interest was screened for transition polymorphisms (i.e. G/A and C/T SNPs) consistent with mutations commonly arising from alkylating agents, including ethylene imine (Griffiths et al. 2000; Verschaeve and Kirsch-Volders 1990). A total of 5242 and 10,738 SNPs with transitions were identified in Chittick and Geebung relative to the Tanjil reference genome, respectively. Of these transitions, 2507 were unique to Chittick and were subsequently compared to the SNPs present in the region of interest in a panel of 43 other resequenced narrow-leafed lupin accessions. Following this comparison, a single G/A SNP was found to be unique to Chittick within the predicted ORF of a gene comprising nine exons. This gene represented one of 40 ORFs annotated by FGENESH within the region of interest (Online Resource 10).

**Fig. 2:**
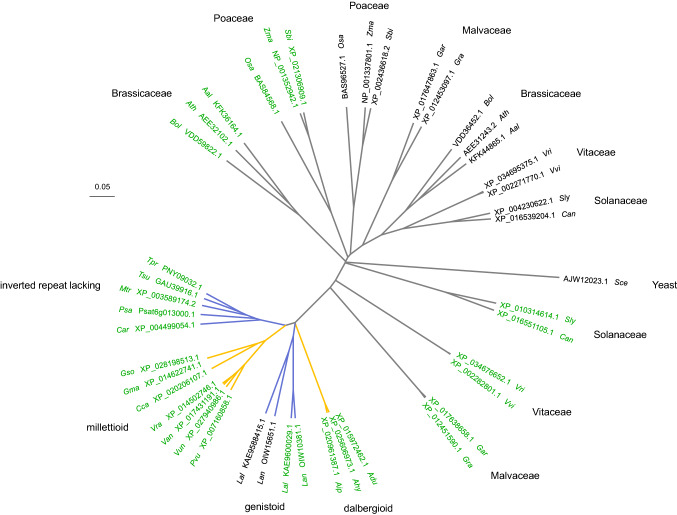
Neighbour-joining phylogenetic tree depicting homology of the Trimethylguanosine Synthase1-like gene family in six angiosperm families and yeast (*Saccharomyces cerevisiae*). Taxa and protein accession IDs are coloured to differentiate eukaryotic (black) and plant-specific (green) homologues. Yellow and purple tree branches distinguish papilionoid clades typically comprising warm-season and cool-season legume species, respectively. Taxa abbreviations and protein accession IDs are fully described in Online Resource 2

The candidate gene containing the unique G/A SNP was identified as *Trimethylguanosine Synthase1-like* (*TGS1*) (*Lup005529.1*, also referred to as *TanjilG_05529;* (Hane et al. 2017)). In contrast to all other eukaryotes, which possess a single *TGS1* orthologue, most diploid plant species thus far appear to contain two *TGS1* homologues within their genomes (Mouaikel et al. 2003). The second *LanTGS1* homologue (*Lup008227.1*, also referred to as *TanjilG_08227;* (Hane et al. 2017)) was located on pseudochromosome NLL-03. Translated sequences from both narrow-leafed lupin homologues (OIW10381.1 and OIW15651.1) shared S-adenosyl-L-methionine-dependent methyltransferase (SAM MTase; IPR029063) and RNA cap guanine-N2 methyltransferase domains (IPR019012), consistent with the methyltransferase function of TGS1 proteins. However, the presence of a WW domain (IPR001202), which is a defining feature of plant-specific TGS1 homologues, was exclusive to OIW10381.1 and indicated that its associated gene *Lup005529.1* is the plant-specific *LanTGS1* homologue. Phylogenetic analysis of several papilionoid legumes and diploid species from five other agriculturally important angiosperm families revealed the two *LanTGS1* homologues still share a very high degree of homology despite this difference. Interestingly, eukaryotic (i.e. non-plant-specific) *TGS1* homologues do not appear to have been conserved beyond the genistoid clade within the papilionoid subfamily of legumes (Fig. 2). Given that plant-specific *TGS1* homologues have roles in mediating chilling tolerance in Arabidopsis (Gao et al. 2017) and regulating apomixis (i.e. asexual reproduction via seeds) in *Paspalum notatum* (Siena et al. 2014; Colono et al. 2019), *Lup005529.1* was considered worthy of further investigation as a candidate vernalisation-related flowering time gene for *efl*.

#### Co-segregation of a transition mutation with the *efl* phenotype

The G/A SNP unique to Chittick in *Lup005529.1* was genotyped in the *F*_2_ and *F*_6_ bi-parental populations and a diverse range of wild and domesticated narrow-leafed lupins to confirm its co-segregation with *efl* phenotype. Within the *F*_2_ population, DNA from the 33 earliest and 32 latest flowering *F*_2_ individuals was combined into two separate bulk samples (Fig. 1a). The early (*efl*-like) bulk returned a homozygous A genotype identical to Chittick. Meanwhile, both alleles were detected in the late (*Efl*-like) bulk. This was consistent with expectations that heterozygous and homozygous G genotypes should exhibit the dominant *Efl* phenotype. Subsequent genotyping of individual *F*_2_ plants within the late bulk confirmed the absence of homozygous A genotypes (Online Resource 4). Within the *F*_6_ RIL population, 86 homozygous A (Chittick-derived), 96 homozygous G (Geebung-derived), and three heterozygous G/A genotypes were produced. In all instances, the genotype of the SNP was consistent with the recombination of DArTseq markers adjacent to the *efl* locus on linkage group CxG_LG14 and perfectly correlated with the qualitative *efl* phenotype-derived marker (Online Resources 1 and 9). Finally, the SNP was perfectly predictive of *efl* phenotype in 40 wild and 49 other domesticated narrow-leafed lupins (Online Resource 5). The rare homozygous A genotype was observed only in Chittick, Wandoo, and the original *efl* mutant N4229, which are the only narrow-leafed lupin accessions with the *efl* mutation (Cowling 1999). Perfect linkage of the unique transition mutation in *Lup005529.1* with *efl* phenotype in three separate genetic and natural narrow-leafed lupin populations warranted further investigation of the plant-specific *LanTGS1* homologue.

#### Exploring impacts of a transition mutation on TGS1 protein function

**Fig. 3:**
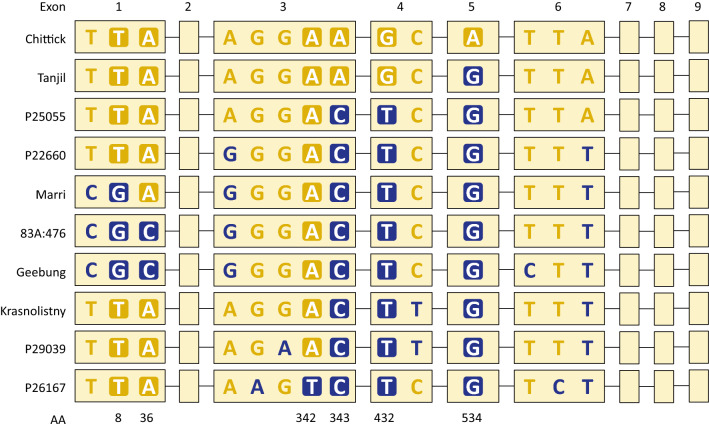
Schematic illustration of the 10 unique single nucleotide polymorphism (SNP) haplotypes observed within the coding sequence (1815 nucleotides) of a plant-specific *Trimethylguanosine Synthase1-like* (*LanTGS1*) homologue (*Lup005529.1*) in narrow-leafed lupin (*Lupinus angustifolius* L.). SNPs have been colour coded such that gold represents the Chittick-like (*efl*) genotype, while blue represents an alternative genotype. Background fills are used to highlight SNPs that create non-synonymous mutations resulting in amino acid (AA) changes. The number beneath each non-synonymous SNP corresponds to the AA residue affected within the translated protein sequence. AA residue 534 corresponds to the Chittick haplotype which has a unique G to A mutation. The reference allele corresponds to the genotype presented in the Tanjil variety haplotype

The full-length predicted CDS and translated protein sequences derived from *Lup005529.1* were closely examined to establish all polymorphisms within the plant-specific *LanTGS1* homologue and determine if any, including the G/A SNP already under investigation, may explain phenotypic differences between *efl* and *Efl* genotypes. A total of 14 SNPs and 10 unique CDS haplotypes were observed among 49 resequenced accessions (Fig. 3; Online Resource 11; MZ274313-MZ274322). Six of the 14 SNPs created non-synonymous mutations in the translated protein sequences, including the candidate causal mutation for *efl* (i.e. the G/A transition unique to Chittick, Wandoo, and N4299), which led to an alanine/threonine substitution at amino acid residue 534. Six unique protein sequences were translated from the CDS haplotypes. Importantly, all three narrow-leafed lupin accessions with the *efl* phenotype (Chittick, Wandoo, and N4229) shared the same CDS haplotype and translated protein sequence, which differed from that of Borre (the variety used to derive *efl* via mutagenesis) and the Tanjil reference genome only by the G/A SNP and associated alanine/threonine substitution (Online Resource 11).

**Fig. 4:**
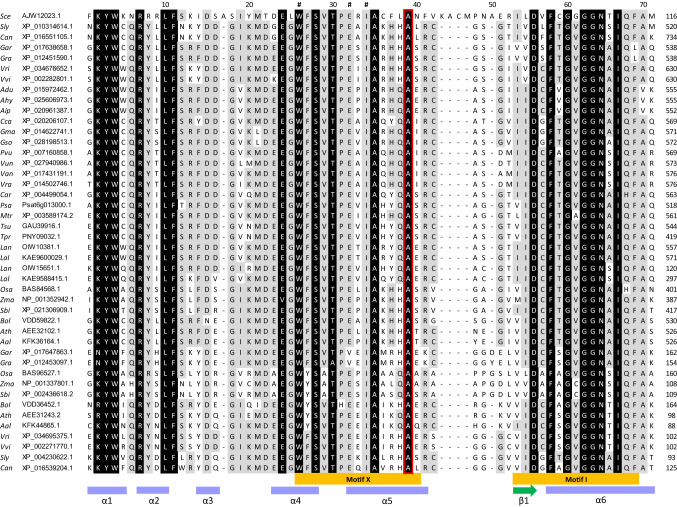
Partial alignment of the S-adenosyl-L-methionine-dependent methyltransferase (SAM MTase) superfamily domain (IPR029063) within *Trimethylguanosine Synthase1-like *(*TGS1*) protein sequences from yeast (*Saccharomyces cerevisiae*) and 29 diploid angiosperms. Shading indicates perfectly (black) and highly (≥ 50%; grey) conserved residues. Conserved motifs within the SAM MTase superfamily of proteins are labelled according to annotations from Mouaikel et al. (2003) and Fauman et al. (1999). Secondary protein structures are indicated by purple cylinders (*α* helix) and green arrows (*β* strands). A red box highlights an alanine residue which is substituted with threonine in the Chittick narrow-leafed lupin variety containing the *efl* mutation for facultative vernalisation requirement and mid-season flowering. Residues with motif X that have been experimentally mutated by Mouaikel et al. (2003) are marked by an “#” symbol. Taxa abbreviations and protein accession IDs are fully described in Online Resource 2

Functional domains and conserved motifs were annotated in the six unique translated protein sequences to determine whether any of the non-synonymous substitutions had potentially compromised protein function. As described earlier, three protein domains were predicted, including (1) a WW domain (IPR001202; amino acids 297–326), (2) an RNA cap guanine-N2 methyltransferase domain (IPR019012; amino acids 541–695), and (3) an S-adenosyl-L-methionine-dependent methyltransferase (SAM MTase) superfamily domain (IPR029063; amino acids 503–660). The latter was the only domain affected by one of the six non-synonymous mutations identified during haplotype analysis. Specifically, the SAM MTase domain overlapped residue 534, which was affected by the candidate causal SNP for *efl* (i.e. the G/A transition). An alanine residue was invariably conserved at this position in eukaryotic and plant-specific TGS1 proteins in yeast and numerous diploid species from the papilionoid legume subfamily and five other angiosperm families (Fig. 4; Online Resource 12). The only observed substitution (alanine to threonine) at this location was in the translated sequence of the Chittick haplotype. The location of this substitution additionally corresponded with motif X, which represented one of nine classical motifs in the Class I SAM MTase superfamily that were annotated in the translated TGS1 sequences (Fig. 4; Online Resource 12; Fauman et al. 1999; Mouaikel et al. 2003).

**Fig. 5:**
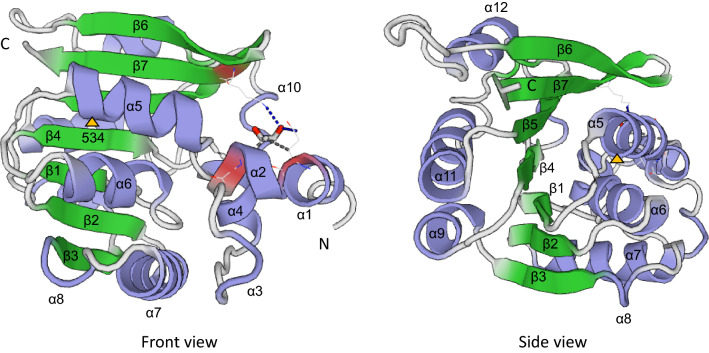
**a** Front and **b** side views of the SWISS-MODEL predicted secondary and tertiary structure of the plant-specific *Trimethylguanosine Synthase1-like *(*TGS1*) protein (OIW10381.1; 717 amino acids) in narrow-leafed lupin (*Lupinus angustifolius* L.). The model represents residues 493–696, which share homology to the human TGS1 template, 3dgh.1.A. Purple helices represent α-helices and green arrows represent *β*-strands, which are numbered. An orange arrow is used to highlight an alanine/threonine substitution at amino acid residue 534, which co-segregates with the *efl* phenotype for mid-late season flowering time and is located within a motif (motif X) involved in substrate binding

3D models of the Tanjil (*Efl*) and Chittick (*efl*) translated sequences were constructed to assess the location of residue 534 relative to secondary and tertiary protein structures and determine whether the alanine/threonine substitution affected protein function. Both models featured classical Class I SAM MTase core folds (Martin and McMillan 2002), which consisted of alternating *β* strands and α helices forming an overall twisted *β* sheet structure (Fig. 5). Four α helices were located at the N terminal of the core fold forming a small subdomain. Additional secondary structures (*α*8, *α*10, *β*5, and *β*7) were present within loops linking the central components of the *β* sheet. Residue 534 was located within the first α helix (*α*5) contributing to the central *β* sheet structure (Figs. 4 and 5). This result was consistent with expectations for the structural placement of motif X in α helices within Class I MTase proteins (Fauman et al. 1999; Mouaikel et al. 2003). Motif X additionally encompassed part of the proceeding α helix (*α*4) and the loop connecting *α*4 and *α*5.

#### Investigating the regulation of a candidate gene under vernalising and non-vernalising conditions

Finally, we explored the expression of *Lup005529.1* in leaves of Chittick and Geebung to learn more about its potential involvement in regulating flowering time within the vernalisation pathway. The relative abundance of *Lup005529.1* transcript was unaffected by vernalisation treatment (*P* = 0.1347) and was stable across all five plant growth stages (*P* = 0.2028). Although the relative expression of *Lup005529.1* was significantly higher in Geebung than in Chittick (*P* = 0.0119), the fold difference between *efl* and *Efl* genotypes was minimal at all times (1.4-fold) (Online Resource 13). The absence of differential expression between parents complemented motif annotation results and was consistent with the hypothesis that the *efl* mutation impacted the function rather than the regulation of the plant-specific *LanTGS1* gene.

## Discussion

The timing of flowering is a crucial adaptive trait affecting the reproductive fitness of wild plants and domesticated crops alike. Vernalisation is one of several important mechanisms used to regulate flowering time in species originating from temperate climates. The objective of this study was to explore the genetic basis and possible molecular role of the *efl* mutation within the vernalisation pathway of narrow-leafed lupin; a Mediterranean winter-annual grain legume. Using 2314 DArTseq molecular markers, we produced a linkage map for an *F*_6_ RIL population comprising 181 lines segregating for *efl* and which was rapidly progressed from the *F*_2_ generation over a period of 13 months using an optimised aSSD method without bias towards early flowering (Croser et al. 2016). This linkage map adds to the growing number of genetic and genomic resources for this species (Singh et al. 2020) and will be useful for resolving inconsistencies in the physical assembly of the narrow-leafed lupin reference genome (Hane et al. 2017), particularly on pseudochromosome NLL-10, which represented 17% of the 781.2 cM map (Table 1). Genetic mapping localised *efl* to a 1.26-cM interval on NLL-10 corresponding to a 267-Kb physical region within an improved genome assembly (Online Resource 7). A single promising candidate gene was identified within this region: a plant-specific *TGS1* homologue, *Lup005529.1*. This is the first time a *TGS1* orthologue has been linked to the vernalisation pathway and flowering time control in plants and consequently represents an important finding.

A single G/A SNP within the plant-specific *LanTGS1* homologue was perfectly predictive of the *efl* phenotype in bi-parental mapping populations and diverse germplasm (Fig. 4; Online Resources 7 and 8). This polymorphism is consistent with mutations arising from ethylene imine treatment, where guanine DNA bases are preferentially alkylated, depurinated, and subsequently vulnerable to random and incorrect replacement with an alternative base during replication of single-stranded DNA (Verschaeve and Kirsch-Volders 1990). Importantly, the SNP was found to create a non-synonymous mutation within a well-conserved motif (motif X) in the SAM MTase protein domain. An alanine residue was perfectly conserved in the eukaryotic and plant-specific TGS1 homologues of 17 papilionoid legumes, 12 diploid species from five other angiosperm families as well as yeast. In stark contrast, the translated plant-specific TGS1 sequence of Chittick (*efl*) had a threonine at residue 534. Together with an absence of differential expression of *Lup005529.1* between Chittick and Geebung, this observation strongly suggested that the substitution arising from the G/A SNP may have altered protein function.

Experimental confirmation that the alanine/threonine substitution in motif X alters the plant-specific LanTGS1 protein function was beyond the scope of this current study; however, functional characterisation of mutations in motif X has previously been performed in yeast (Mouaikel et al. 2003). In yeast, *ScTGS1* is responsible for post-transcriptional maturation of snRNA and snoRNAs cap structures, which it achieves by hypermethylating the m^7^G cap of these RNA species to m_3_G (i.e. trimethylguanosine) caps. The mature snRNA and snoRNAs then go on to play roles in processing immature mRNA and rRNAs, respectively. Knock-out and loss-of-function mutations in *SceTGS1* inhibit the hypermethylation and correct splicing of immature snRNA and snoRNA, which manifests in a cold-sensitive phenotype and abnormal development under cool temperature (Mouaikel et al. 2002). A similar sensitivity to cold ambient temperature and defects to vegetative and reproductive organs result from mutations in the plant-specific *AthTGS1* homologue in Arabidopsis (Gao et al. 2017). Mouaikel et al. (2003) demonstrated that the α helix structure containing motif X participates in forming the m^7^G cap substrate binding pocket of SceTGS1 by targeted substitution of isoleucine for arginine at residue 175 (one of three residues assessed within motif X, as indicated in Fig. 4). This substitution prevented restoration of normal development in *Scetgs1* loss-of-function mutants and was correlated with the absence of m_3_G cap structures in snRNAs and snoRNAs. Mouaikel et al. (2003) proposed that the side chain associated with arginine physically blocked the binding site for m^7^G caps and therefore inhibited methyltransferase activity of the mutated Scetgs1 protein. Further research will be required to determine whether the alanine/threonine substitution at residue 534 follows precedent and similarly affects the methyltransferase activity of the plant-specific LanTGS1 protein and, as a result, affects vernalisation requirement and flowering time.

It remains unclear exactly how *efl* affects the vernalisation response in narrow-leafed lupin. The mutation was originally proposed to reduce the requirement for vernalisation (Gladstones 1977, 1982). Subsequent research by Landers (1995) indicated this was achieved through the creation of a facultative rather than obligate requirement for vernalisation. However, the same study also brought into question whether *efl* may instead increase the temperature threshold at which vernalisation operates. White lupin (*L. albus*) sets precedence for such a mechanism as plant development in selected vernalisation-responsive genotypes (e.g. variety Ultra) is reportedly accelerated under temperatures as high as 17 °C in as little as 10 days (Clapham and Willcott 1995; Huyghe 1991). The identification of *Lup005529.1* as a candidate gene for *efl* may offer further support for this latter hypothesis, as mutations in *TGS1* affect thresholds at which development is impaired during cold treatment. For example, Arabidopsis *tgs1* mutants develop minor defects in their leaves and flowers when grown at 10 °C but develop more severe abnormalities that inhibit viable seed production at 5 °C (Gao et al. 2017). Similarly, yeast *tgs1* mutants are able to grow, albeit slowly, at 23 °C but are fully incapable of doing so once ambient temperature is reduced to 16 °C (Mouaikel et al. 2002).

There are positive implications for narrow-leafed lupin pre-breeding should *efl* indeed reduce the temperature and/or duration threshold at which vernalisation is satisfied in this species. Early flowering, vernalisation-unresponsive varieties with the *Ku* and *Jul* alleles are now almost exclusively sown for all agricultural grain production in Australia and Northern Europe. However, the dominance of both alleles over most other genetic variation for flowering time (Boersma 2007) and the central role of their underlying gene (*LanFTc1*) within the genetic network for floral initiation reduces the ability for breeders to manage other minor alleles for phenology in the absence of marker-assisted selection. In fact, the *efl* mutation would not have been discovered if the mutation occurred in a *Ku* or *Jul* variety due to their dominant early flowering genotypes. Late-flowering *ku* varieties of narrow-leafed lupin require a strong vernalisation period which may not be met every season, at least within mild winter climates. The milder facultative vernalisation requirement of *efl* varieties could be a major advantage in such situations. Alternatively, new mutations within *Lup005529.1* via gene editing or TILLING may enable the temperature threshold for vernalisation to be sufficiently increased such that *ku* varieties are better adapted to warmer climates. This would ultimately create an opportunity for other flowering time loci to be integrated in breeding and establish a wider range of phenologies in elite varieties.

As the candidate SNP was exclusively associated with the *efl* phenotype in this study, it is now possible for a perfect molecular marker to be designed for future genotypic selection (marker-assisted selection) of *efl* within breeding programmes. Genotypic selection would greatly facilitate selection for subtle flowering time alleles, such as *efl*, which are at risk of being lost during the early stages of phenotypic selection due to their recessive inheritance, particularly if breeding lines are evaluated in environments or seasons where these alleles do not provide an adaptive advantage.

## Supplementary Information

Below is the link to the electronic supplementary material.

Supplementary file1 (XLSX 16901 KB)

## Data Availability

All data relating to this study are freely available within the Supplementary Information and upon request to the corresponding author. gDNA and protein sequences have been deposited to NCBI repositories, including GenBank and SRA archives, and accession numbers are provided within text. Seed produced from the *F*_6_ RIL population in this study have been deposited into the Australian Grains Genebank (AGG), Horsham, Victoria.
